# A cross-sectional study to explore the relationship between the technology acceptance model and burnout and depression among pharmacists working with a pharmacy robotic dispensing system

**DOI:** 10.3389/fpsyg.2024.1436518

**Published:** 2025-01-17

**Authors:** Amna Ibrahim Alshamsi, Mariam AlHarthi, Hessa AbdulQader, Pramod Chhabrani, Safa Ahmed, Muna Almansoori

**Affiliations:** ^1^Emirates Health Services (EHS), Dubai, United Arab Emirates; ^2^Al-Amal Psychiatric Hospital, Dubai, United Arab Emirates; ^3^Al Kuwait Hospital Dubai, Dubai, United Arab Emirates; ^4^Al Dhaid Hospital, Sharjah, United Arab Emirates

**Keywords:** pharmacy robotic dispensing system, technology acceptance model, burnout, depression, pharmacists

## Abstract

**Objectives:**

This study compares hospitals using a pharmacy robotic dispensing system (RPDS) with those using manual dispensing systems regarding burnout and depression among pharmacists in Emirates Health Services (EHS) hospitals. Furthermore, this study aims to bridge the gap in the literature concerning the relationship between burnout and the technology acceptance model (TAM).

**Methods:**

A cross-sectional survey was conducted to determine whether burnout and TAM differed between hospitals with RPDS and those with manual dispensing system. The study was carried out in ten hospitals governed by the EHS.

**Results:**

A total of 256 respondents completed the survey. Burnout and depression levels among pharmacists working with RPDS did not differ significantly from those using manual dispensing systems. However, the median of personal burnout levels in female pharmacists (Mdn = 50) differed significantly from those using manual dispensing systems (Mdn = 25; U = 3497.5, *z* = −7.8, *p* < 0.001, *r* = −0.49). In contrast, male pharmacists exhibited higher levels of technology acceptance (U = 11,357, *z* = 5.58, *p* < 0.001, r = 0.35; U = 10,391, *z* = 4.0, *p* < 0.001, *r* = 0.25).

**Conclusion:**

This study explored the differences in burnout, depression levels, and TAM among employees working in public hospitals in the United Arab of Emirates. Overall, automation had both positive and negative effects on workplace stressors experienced by pharmacy staff.

## 1 Introduction

### 1.1 Pharmacy robotic dispensing system

The use of advanced technology in the healthcare sector has increased. Robots have the potential to support various aspects of healthcare and assist humans in diverse environments. Their applications span across multiple specialties and settings. Morgan et al. (2022) reviewed the use of robots in healthcare, identifying their roles and deployment in various clinical settings, such as surgical theaters to rehabilitation units, hospital wards, and inpatient pharmacies. Robotic systems aim to enhance patient experiences, increase satisfaction, and reduce dispensing errors (Boyd and Chaffee, 2019). While advanced technology is essential in healthcare, there is limited evidence regarding its impact on the mental health of healthcare professionals, particularly concerning burnout and depression. This study explores the relationship between work-related well-being and the acceptance of advanced technology and is among the first to highlight the history of robotic use in healthcare.

The first documented robot-assisted surgical procedure occurred in 1985. Since then, technological advancements have rapidly enhanced the capabilities of robots (Kyrarini et al., 2021). Healthcare organizations worldwide are investing in expensive technologies to improve care delivery and patient safety. The Emirates Health Services (EHS) introduced a pharmacy robotic dispensing system (RPDS) in five major hospitals to expand the pharmaceutical service automation. RPDS are autonomous systems designed to dispense medications to inpatient and outpatient departments and manage medication storage and inventory (Boyd and Chaffee, 2019). By reducing the need for human intervention, these systems aim to minimize the risk of human error (James et al., 2013). However, studies have found that some staff members underuse or misuse these technologies (Holden and Karsh, 2010).

The RPDS mimics human behavior through algorithms that map and analyze the environment. These algorithms allow the system to adapt to its surroundings, which is crucial for its effectiveness. Accurate mapping is essential for the practical applications performed by pharmacy robots. Consequently, RPDS is set to influence the evolution of traditional medical dispensing practices (Barrett et al., 2012; James et al., 2013). A RPDS is considered an intelligent agent, either virtually or mechanically, operating under human supervision through automation. Recent advancements have increased its applications, including in Middle Eastern facilities. However, empirical studies on the impact of RPDS on employees’ well-being and technology acceptance with EHS hospitals is lacking.

Healthcare providers are constantly challenged to engage in advanced technical functions (Shanafelt et al., 2015). Many healthcare organizations have introduced automation tools, including RPDS, to address medication incidents and deficiencies. Although automation is claimed to improve working conditions and ease prescription processing, some technicians still face physical and mental challenges (James et al., 2013; Boyd and Chaffee, 2019). A case study in the United Kingdom (UK) aimed to determine the effect of installing a RPDS and reported that automation has enabled the expansion of pharmacists’ roles (James et al., 2013). James et al. (2013) suggested that automation positively impacted staff experiences related to stress, illogical workload allocation, work-life balance, and overall working conditions.

Researchers are increasingly interested in exploring connections between technology and mental health. Pinto et al. (2024) conducted a systematic review to investigate the relationship between emerging workplace technologies and employee mental health, focusing on burnout. Their findings revealed that burnout among staff is linked to inadequate training and feelings of insecurity when using advanced technology. Additional studies have shown that health information technology, especially electronic medical records, can significantly contribute to clinician burnout (Wu et al., 2021). However, there is limited understanding of the relationship between RPDS and staff burnout.

Few studies have assessed the relationship between the Technology Acceptance Model and burnout and depression among pharmacists working with a pharmacy robotic dispensing system. We believe our study aims to contribute to this growing area of research through its novel examination of the role of TAM in predicting burnout and depression amongst pharmacists working with RPDS in the United Arab Emirates (UAE).

### 1.2 Burnout

Job burnout occurs in every occupation. In healthcare, burnout has been studied more frequently among physicians and nurses; some studies have shown that it can also affect pharmacists (Prasad-Reddy et al., 2021). Burnout is characterized by feelings of emotional exhaustion and depersonalization (Maslach and Jackson, 1981). Previous research has demonstrated that organizational risk factors, such as increased or decreased workload, lack of job control, ineffective reward systems, and insufficient social interaction with colleagues and supervisors, can affect staff burnout levels (Maslach et al., 2008). Hobfoll and Shirom (2001) proposed a mechanism for burnout through the Conservation of Resources Theory. They suggested that burnout occurs when individuals excessively invest their resources without receiving sufficient returns. Individuals become cautious with future resource allocation when there is a lack of investment return. This may lead to distancing from the newly added technology or developing negative attitudes toward it.

Many researches have shown that workload is associated with work-related stress, which is recognized as a significant psychosocial hazard (Harvey et al., 2017). Understanding this relationship is essential, as workers may face an overload or underload of tasks affecting their stress level. Additionally, undergoing organizational changes can negatively impact employees’ mental health (Harvey et al., 2017). A change in the work system includes introducing new and different work aspects designed to enhance productivity or improve services within the workplace (Murphy et al., 2002). The addition of new technology (i.e., RPDS) can increase pharmacists’ experience of stress and burnout. Indeed, organizational culture can influence burnout. Negative culture can be stressful, mainly when managers or supervisors do not communicate appropriately with subordinates. Evidence suggests organizational culture could affect individuals’ health and well-being (Cox and Howarth, 1990).

Burnout is associated with higher job turnover, reduced productivity, and quality concerns regarding patient safety and satisfaction. A growing body of literature has investigated the impact of burnout on pharmacists’ well-being and patient safety (Babal et al., 2020; Holden and Karsh, 2010; Ortmeier and Wolfgang, 1991). For example, Holden et al. (2010) found that external demands, including interruptions, divided attention, and increased work pace, negatively impacted medication safety and employee well-being outcomes. Therefore, investigating the level of burnout among pharmacists working in EHS is essential. This study examines the relationship between burnout and pharmacists’ acceptance of the RPDS using the technology acceptance model (TAM) (Davis, 1989). Based on our literature review, no previous study in the UAE has explored burnout among pharmacists working with a RPDS compared to those using manual dispensing systems.

The rapid development of healthcare technologies necessitates more professional devotion, leading to burnout. Therefore, the fatigue and complexity of newly introduced technology (i.e., RPDS) may result in burnout among pharmacists. Previous studies on TAM and burnout have shown that TAM is linked to staff’s ability to perform their work responsibilities without unnecessary complexity, potentially reducing their sense of burnout (Davis, 1989; Kamel, 2024). Kamel (2024) examined the impact of TAM on job burnout among employees in travel agencies. The study showed that TAM moderates the relationship between technology-related demands and burnout. Consequently, pharmacists with high burnout levels are less likely to accept new technologies. Therefore, our primary objective is to compare hospitals using RPDS with those using manual dispensing systems regarding burnout and depression among pharmacists in EHS.

Sex also plays a role in burnout. It is reported that females are more likely than males to experience burnout and exhaustion at work (Artz et al., 2022). Female workers often face potential conflicts between family and work, leading to burnout and reduced job and life satisfaction. Therefore, this study is vital because it assesses the role of sex in working with manual dispensing systems in the UAE, a Middle Eastern country.

Hypothesis 1 (H1): *Pharmacists working with RPDS have different burnout levels compared to pharmacists working with manual dispensing system.*

Hypothesis 2 (H2): *Female pharmacists have different burnout levels compared to male pharmacists working at EHS hospitals.*

### 1.3 Technology acceptance model

Despite advancements in healthcare technology, healthcare organizations continue to face challenges of underutilization. In the 1980’s, Davis (1989) developed the technology acceptance model (TAM) to reliably predict the actual use of a new technology. He hypothesized that users’ attitudes towards a new technology will determine if an individual will use or reject a technology (Holden et al., 2010). By understanding the factors that influence employees’ intention to use new technologies, organizations can then manipulate these factors to increase acceptance and use of those technologies. TAM has two main variables, perceived ease of use and perceived usefulness, which are precursor factors affecting technology acceptance (Granić and Marangunić, 2019).

Perceived ease of use (PEOU) refers to the belief a person has that using a new system would be free of effort (Davis, 1989). PEOU evaluates different aspects, including whether the new technology is easy to use, clear and understandable, and capable of quickly training staff (Holden et al., 2010). Perceived usefulness (PU), in contrast, is the extent to which staff think using the system will enhance their performance at work (Davis, 1989; Venkatesh and Davis, 2000). PU was typically assessed by inquiring about the health technologies’ usefulness for specific tasks, their impact on productivity, and their effect on job significance (Holden et al., 2010). Perceived usefulness is a strong determinant of usage intention and can be influenced by other factors, including staff burnout. More research highlighting the role of staff burnout in the acceptance of new technologies is needed.

In healthcare, most studies focused on the acceptance of using electronic medical records (EMR) (Simon et al., 2009). Also, the literature on TAM pays particular attention to learning technology, such as blended and virtual learning (Granić and Marangunić, 2019). Previous studies have suggested introducing new technologies can create unexpected tasks for pharmacists (Holden et al., 2010; James et al., 2013; Boyd and Chaffee, 2019; Siska and Tribble, 2011).

Some studies have reported that RPDS improves staff satisfaction and work experience while reducing stress (Hogan et al., 2020). Other studies found adverse outcomes associated with such technologies, including challenges in implementing RPDS (Boyd and Chaffee, 2019). Therefore, this study investigates the factors influencing hospital pharmacies’ acceptance of RPDS using the technology acceptance model (TAM) (Venkatesh and Davis, 2000). Therefore, this study compares pharmacists working with RPDS and those working with manual dispensing systems based on PEOU and PU. We operationalized PEOU in RPDS as easy to use, clear, and understandable, whereas PU is operationalized as helpful in completing RPDS tasks. Our hypothesis is as follows:

Hypothesis 3 (H3): *Pharmacists working with RPDS have different PEOU and PU levels compared to pharmacists working with manual dispensing system.*

Finally, we hypothesized that burnout negatively correlates with TAM. Although automation improves working conditions and eases prescription processing, some technicians continue to experience physical and mental demands (James et al., 2013). Based on this information, our final hypothesis is as follows:

Hypothesis 4 (H4): *Burnout is correlated with TAM factors (i.e., perceived ease of use and perceived usefulness).*

## 2 Methods

### 2.1 Study design, settings, and participants

A cross-sectional survey was used to examine whether burnout and TAM differed between hospitals with RPDS and those with a manual dispensing system. The survey also investigated the relationships between burnout, acceptance of RPDS, and sex of participants. The study was conducted in ten hospitals governed by the EHS. The sample included five hospitals with RPDS and five with a manual dispensing system. Hospitals with manual dispensing systems were selected because they were similar to those with RPDS in size and scope of service. These hospitals were selected to represent key aspects of the changes in the EHS that the study aimed to capture. Ethical approval was granted by the MOHAP (MOHAP/DXB-REC/MMM/No.53/2023).

Data were collected from pharmacists in hospitals with RPDS, including Fujairah (MFH), AL Qassimi (AQH), AL Qassimi Women and Children (AQW), Ibrahim Bin Hamad and Obaidullah (MOH), and Abdalla Bin Omran (MOW) hospitals. Furthermore, pharmacists in hospitals with a manual dispensing system included Dibba AL Fujairah (MDH), Khorfakkan (MKF), Saqr (MSQ), Kalba (MKH), and AL Dhaid (MAD) hospitals.

Access to participants was gained by approaching staff working in the pharmacy department at the EHS headquarters and sending them direct invitations to avoid coercion. The email invitation outlined the aims of the study. Invitation were sent by a co-investigator to all pharmacists with a link to the online survey, which included the participant information sheet, consent form, demographic form, and the questionnaire. Reminder emails were sent weekly for four weeks to encourage participation. Data collection started on October 1, 2023, and ended on November 1, 2023.

The sample size was calculated using Statulator, an online tool. Two independent means were compared, determining the sample size based on the effect size (*ρ*), power (i.e., the chance of getting a significant result), and the significance level (*α*) required in the study. Considering that the study design involved a mean difference, the sample size was calculated using an independent t-test average model. The statistical power chosen was 0.80 for a medium effect size (ρ) of 0.3 with a significance value (α) of 0.05. The required sample size was 244 patients (122 in each group). For example, to detect an actual difference in means between RPDS and manual dispensing systems, 122 participants from hospitals with RPDS and 122 participants from hospitals with manual dispensing systems were needed. Participation was voluntary, and pharmacists had to work under the EHS and be at least 18 years old.

### 2.2 Measures

As the cross-sectional study aimed to compare burnout and TAM (i.e., perceived ease of use and perceived usefulness) in pharmacists working with RPDS and manual systems, relevant questionnaire items were informed by a literature review and gap analysis. The questionnaire attempted to measure the constructs experienced by staff in hospitals with RPDS or manual dispensing systems. Self-report questionnaires have been widely used to measure work-related risks due to their cost effectiveness. All necessary items were reverse coded in accordance with the author’s suggestions for each scale.

#### 2.2.1 Burnout

Burnout was measured using the Copenhagen Burnout Inventory (CBI) (19 items) (Kristensen et al., 2005). The study included a personal burnout construct (six items), work-related construct (seven items), and patient-related construct (six items). Personal burnout measures psychological exhaustion experienced by individuals without a specific cause. Items were measured using a Likert scale ranging from a high score of 100 (always) to a low score of 0 (never or hardly ever). The reliability of the personal burnout scale was 0.92.

#### 2.2.2 Patient health questionnaire-9

The PHQ-9 is a self-administered screening tool used to assess the severity of depressive symptoms. Unlike other depression scales, the PHQ-9 includes nine items based on the Diagnostic and Statistical Manual of Mental Disorders, 4th edition (DSM-IV) for Major Depressive Disorder. The questionnaire assessed how often the subjects were disturbed by any of the nine items during the preceding two weeks. Each item of the PHQ-9 was scored on a scale of 0 to 3 (0 = not at all, 1 = several days, 2 = more than a week, 3 = nearly every day). The PHQ-9 total score ranges from 0 to 27 (scores of 5–9 indicate mild depression; 10–14, moderate depression; 15–19, moderately severe depression; ≥ 20, severe depression) (Kroenke et al., 2001).

#### 2.2.3 Technology acceptance model

TAM consists of two major constructs, perceived ease of use (PEOU) and perceived usefulness (PU), which assess attitudes toward technology and its actual use (Shamsi et al., 2021; Venkatesh and Davis, 2000). TAM was adapted from RPDS (Hogan et al., 2020). Perceived usefulness (PU) was measured using four items, such as, “Using the robot would enhance my efficiency.” PEOU was assessed with items such as, “Learning to use the robot will be easy for me.” For both constructs, participants responded on a five-point scale ranging from strongly disagree (1) to strongly agree (5).

#### 2.2.4 Demographic data

The study included demographic variables such as participant age (numeric variable), sex (binary variable), nationality, and job category.

#### 2.2.5 Data analysis

The questionnaire items were presented to participants in the official languages, including Arabic and English. A reliability test (i.e., Cronbach’s alpha) was performed using SPSS Statistics software (version 28). Before testing the hypotheses, several tests were conducted to assess the suitability of the assumptions for the *t*-test and Pearson’s correlation. Although the variables are continuous and randomly sampled from a population, the underlying assumption of equal population variance was not met (Ruxton, 2006). A series of histograms was generated to assess the normality of the distribution. Deviations from normality were observed, as the bell-shaped curve was not normally distributed for the dependent variables. Outliers were identified using a scatterplot and were present in the plots. Missing data were treated using the listwise deletion method because data imputation could introduce bias, potentially affecting relationships between variables. Accordingly, the listwise deletion method was chosen to minimize loss of data (Hughes et al., 2019). Therefore, non-parametric tests including Mann–Whitney (*U*), median (Mdn), and Spearman correlation were conducted to test the study hypotheses (Field, 2018).

## 3 Results

A total of 256 surveys were completed during the data collection phase. Most respondents were male, accounting for 55.1% (*n* = 141). Furthermore, 52.7% (*n* = 135) of the respondents worked in hospitals with RPDS, whereas 47.3% (*n* = 121) worked in hospitals with manual dispensing systems. Of the respondents, 31.3% were in the age group 31–35 years. Table 1 displays the frequency table for the demographic data.

**Table 1:** Frequency table of the demographic variables.

Demographic variable		Frequency (*n*)	Percent (%)
Gender	Female	115	44.9
	Male	141	55.1
Working with RPDS	No	121	47.3
	Yes	135	52.7
EHS facility	MOW	22	8.6
	MSQ	14	5.5
	AQH	39	15.2
	AQW	16	6.3
	MFH	9	3.5
	MOH	49	19.1
	MAD	21	8.2
	MDH	27	10.5
	MKH	24	9.4
	MKF	35	13.7
Age group	<=25	12	4.7
	26–30	31	12.1
	31–35	80	31.3
	36–40	55	21.5
	41–45	44	17.2
	46–50	13	5.1
	51–55	11	4.3
	56–60	10	3.9
Profession	Assistant Pharmacist	54	21.1
	Other	5	2
	Pharmacist	138	53.9
	Principal Pharmacist	2	0.8
	Senior Pharmacist	16	6.3
	Specialist Clinical Pharmacist	26	10.2
	Technician	15	5.9
Shift duties	No	44	17.2
	Yes	212	82.8
Job contract	Contracted services	15	5.9
	EHS employee	241	94.1
Years of experience	< 5 years	40	15.6
	> 10 years	119	46.5
	5–10 years	97	37.9

All scales showed acceptable internal reliability (personal burnout, *α* = 0.92; work-related burnout, α = 0.91; patient-related burnout, α = 0.83; depression (PHQ-9), α = 0.87; perceived ease of use, α = 0.80; perceived usefulness, α = 0.92) (Table 2).

**Table 2:** Correlation matrix for the constructs of TAM and CBI and depression.

Variable	Mean	SD	α	1	2	3	4	5	6
1. Total perceived usefulness	12.7	6.6	0.92	1					
2. Total perceived ease of use	9.9	4.6	0.79	0.64**	1				
3. Total patient health 4. questionnaire	4.1	4.7	0.86	−0.35**	−0.25**	1			
4. Average personal burnout	35.4	23.5	0.92	−0.42**	−0.28**	0.66**	1		
5. Average work-related burnout	29.2	22.8	0.91	−0.36**	−0.21**	0.69**	0.82**	1	
6. Average patient-related burnout	19.1	17.7	0.83	−0.27**	−0.12	0.50**	0.55**	0.67**	1

** Correlation is significant at the 0.01 level (2-tailed).

**Figure 1:**
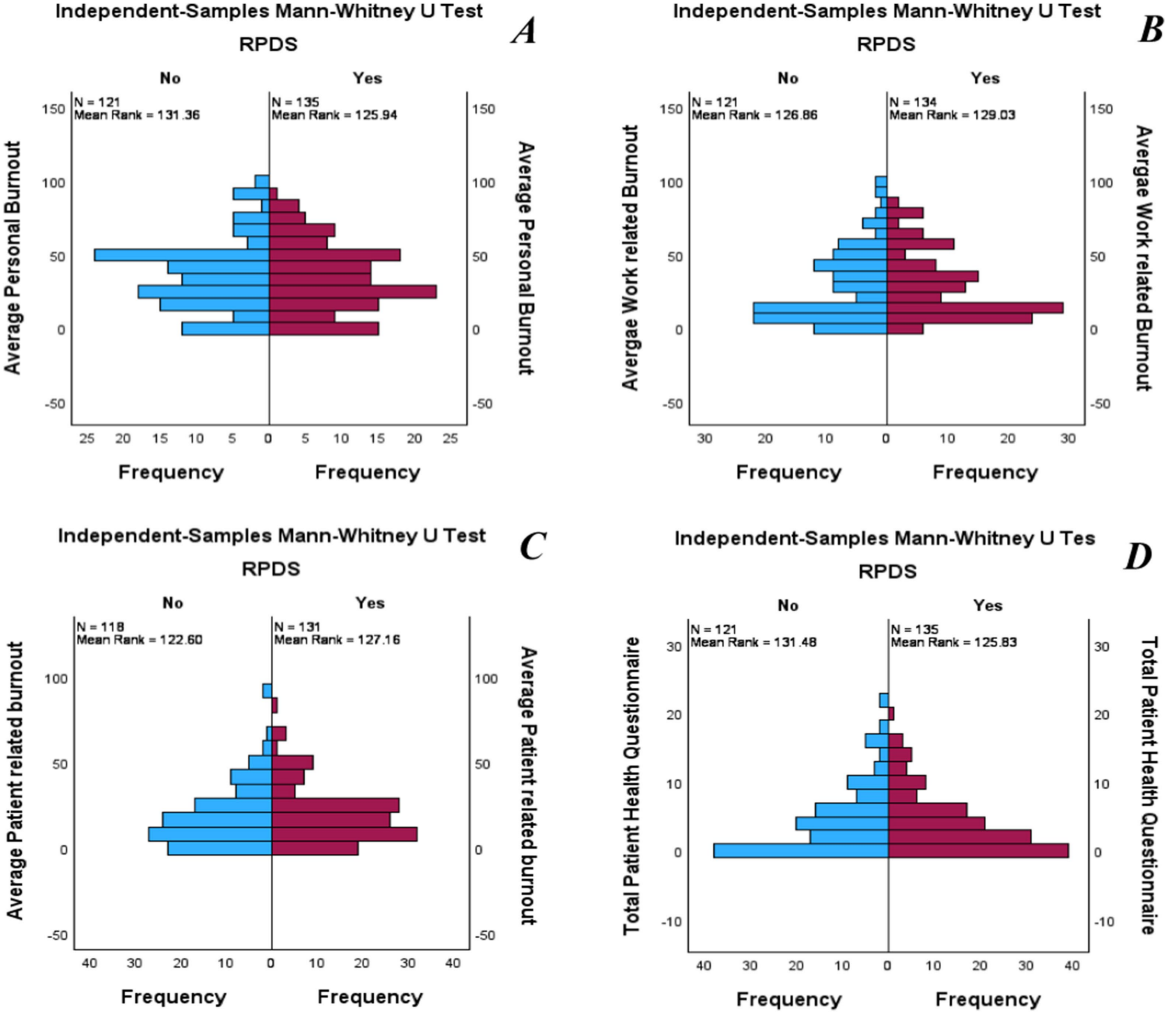
Independent-sample Mann–Whitney U test for burnout and depression level in pharmacists working RPDS and manual dispensing system. **(A)** Shows the difference in personal burnout; **(B)** Shows the difference in work-related burnout; **(C)** Shows the difference in patients-related burnout; and **(D)** Shows the difference in depression.

To test the first hypothesis (H1), an independent Mann–Whitney U test was conducted. Burnout and depression levels in pharmacists working with RPDS did not differ significantly from those in pharmacists working with manual dispensing systems. Personal burnout in RPDS users (Mdn = 33.3) did not differ significantly from that in manual dispensing users (Mdn = 33.3), U = 7,822, z = −0.585, *p* = 0.558, r = −0.037. Work-related burnout in RPDS users (Mdn = 21.43) did not differ significantly from that in manual dispensing users (Mdn = 21.43), U = 8245.5, z = 0.236, *p* = 0.813, r = 0.015. Patient-related burnout among RPDS users (Mdn = 16.6) did not differ significantly from that in manual dispensing users (Mdn = 16.6), U = 8012.5, z = 0.503, *p* = 0.615, r = 0.032. Finally, depression in RPDS users (Mdn = 3) did not differ significantly from that in manual dispensing users (Mdn = 2), U = 7807.5, z = −0.618, *p* = 0.536, r = −0.039. These results reject Hypothesis 1 (Figure 1).

However, when testing the technology acceptance model (H3), perceived usefulness in RPDS users (Mdn = 16) differed significantly from that in manual dispensing users (Mdn = 12), U = 6257.5, *z* = 3.6, *p* < 0.001, *r* = 0.23, thus rejecting the null hypothesis. Perceived ease of use in RPDS users (Mdn = 12) did not differ significantly from that in manual dispensing users (Mdn = 11), U = 5,287, *z* = 1.44, *p* = 0.15, *r* = 0.09.

**Figure 2:**
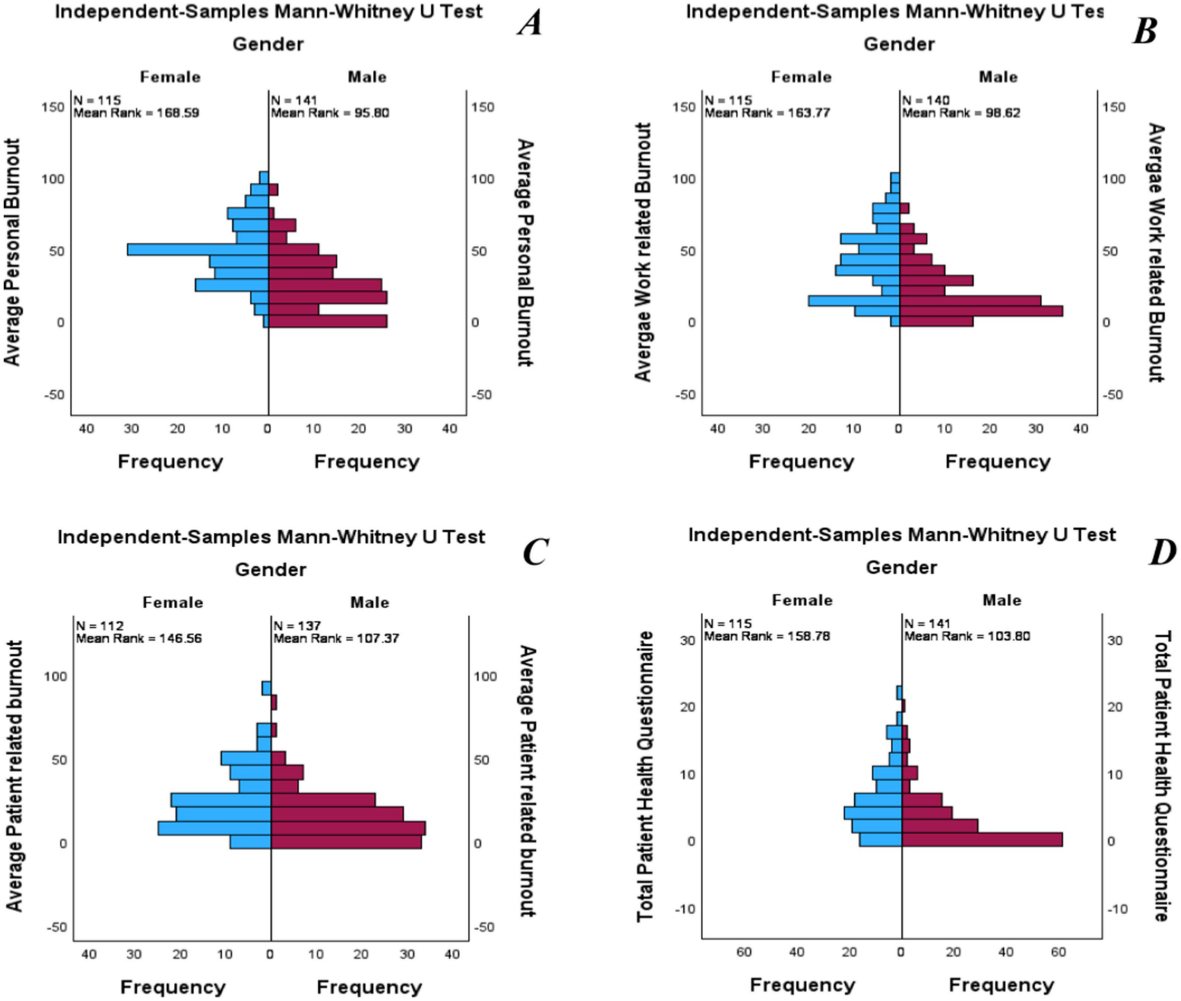
Independent-sample Mann–Whitney U test for burnout and depression level in gender. **(A)** Shows the difference in personal burnout; **(B)** Shows the difference in work-related burnout; **(C)** Shows the difference in patients-related Burnout; and **(D)** Shows the difference in depression.

Furthermore, differences in burnout and depression between sexes were tested to address the second hypothesis (H2). Personal burnout levels in females (Mdn = 50) differed significantly from those in males (Mdn = 25), U = 3497.5, *z* = −7.8, *p* < 0.001, *r* = −0.49. Work-related burnout levels in females (Mdn = 39.29) differed significantly from those in males (Mdn = 14.29), U = 3,937, *z* = −7.03, *p* < 0.001, *r* = −0.44. Patient-related burnout levels in females (Mdn = 20.83) differed significantly from those in males (Mdn = 12.5), U = 5,257, *z* = −4.29, *p* < 0.001, *r* = −0.27. Depression levels in females (Mdn = 5) differed significantly from those in males (Mdn = 1), U = 4,625, *z* = −6.0, *p* < 0.001, *r* = −0.38 (Figure 2).

**Figure 3:**
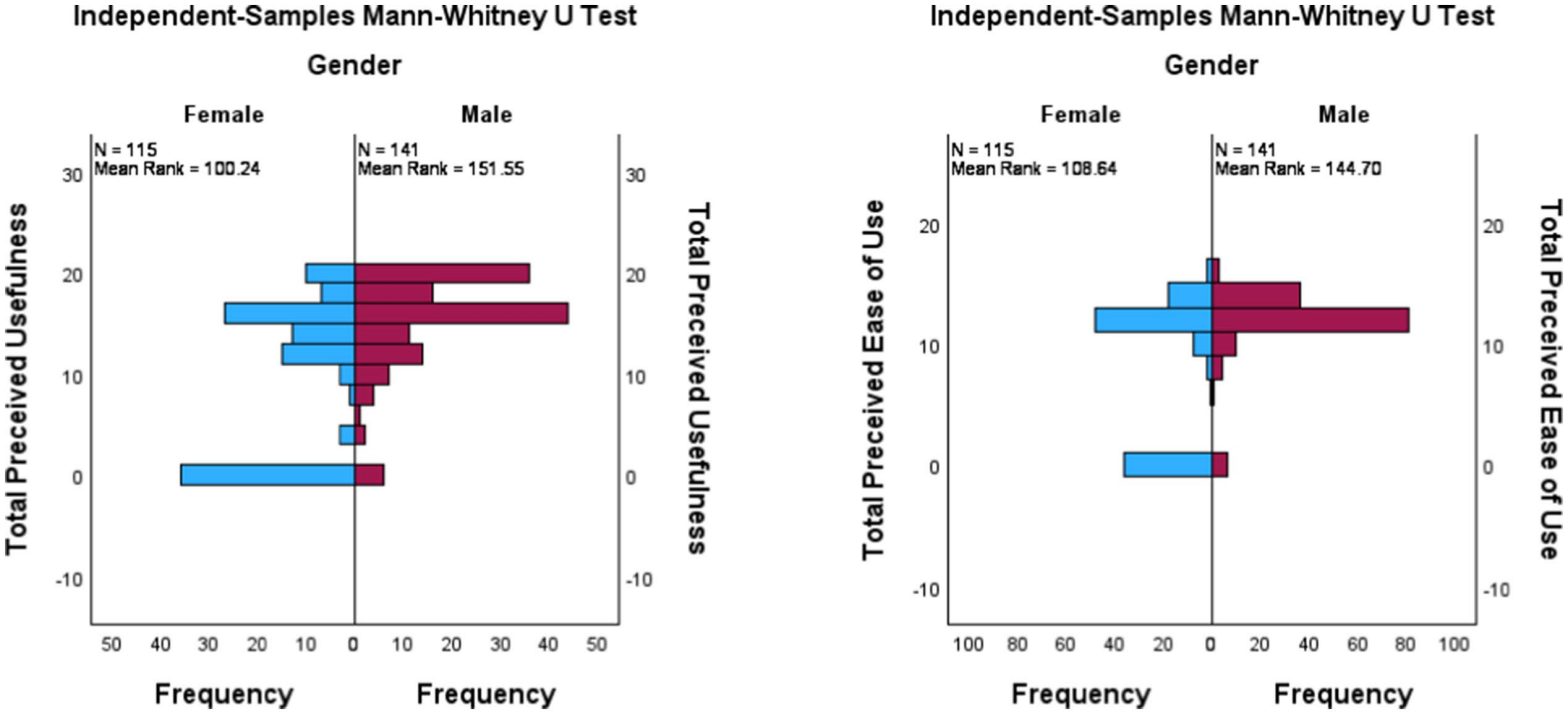
Independent-sample Mann–Whitney U test for TAM. The left graph shows the difference in perceived usefulness. The right graph shows the difference in perceived ease of use.

In contrast, male pharmacists reported higher levels of technology acceptance. Perceived usefulness in males (Mdn = 16) differed significantly from that in females (Mdn = 12), U = 11,357, *z* = 5.58, *p* < 0.001, *r* = 0.35. Perceived ease of use in males (Mdn = 12) also differed significantly from that in females (Mdn = 12), U = 10,391, *z* = 4.0, *p* < 0.001, *r* = 0.25 (Figure 3).

## 4 Discussion

This study aimed to compare hospitals using RPDS with those using manual dispensing systems regarding burnout among pharmacists. The use of RPDS has substantially increased over the past few years. However, empirical studies on the EHS are yet to be conducted to assess the impact of RPDS on pharmacists’ mental health. In this cross-sectional study, based on the responses received (*n* = 256), the majority of HCPs were male (55.1%), pharmacists (53.9%), and aged between 31 and 35 years (31.3%). An unequal ratio of males to females is common in the healthcare sector.

These findings suggest that the median levels of personal burnout, work-related burnout, patient-related burnout, and depression are significantly higher in females. However, such differences were not observed when comparing these variables across the different dispensing systems; and thus, the first hypothesis was not supported. Therefore, the implementation of automation should be accompanied by the identification and rectification of occupational stressors to ensure a balanced work environment. Factors such as workload management, work-life balance, and logical workload allocation should be addressed (James et al., 2013).

Additionally, TAM, which includes perceived usefulness and perceived ease of use, was significantly higher among pharmacists working with RPDS. This finding supports the work of Hogan et al. (2020), who identified the factors influencing staff acceptance of robotic pharmacies. Furthermore, male pharmacists showed lower levels of burnout and higher acceptance of technology than female pharmacists. This indicates that female pharmacists may be less inclined to adopt technology due to their higher levels of burnout (Teo et al., 2015).

The second objective was to address the gap in the literature on the relationship between burnout and TAM. The study found that factors related to TAM (i.e., perceived usefulness and perceived ease of use) were significantly negatively associated with burnout and depression levels, supporting the third hypothesis (H3). These results align with Kamel’s (2024) findings, which highlight that technology overload and complexity contribute to burnout. Some users encounter new technologies and platforms that are complex and unfamiliar, resulting in system failures and reduced employee performance. To address these challenges, managers should support employees by providing effective guidance on using new technologies. This includes offering autonomy for workload management and sufficient training.

Although this study yielded significant findings, it has limitations. First, we could not conclude the causality of the reported relationship between EHS staff burnout and acceptance of RPDS. This study used a cross-sectional design with a total sample of 256 pharmacists, meaning the findings apply only to pharmacists working in the hospitals selected for this study. Future studies should explore the effects of RPDS on staff and the work environment using longitudinal designs. We could not evaluate the impact of specific psychosocial work hazards on pharmacists and burnout levels. For instance, female workers exhibited higher burnout levels than their male counterparts, indicating a potential work–family conflict. Additionally, further assessments of the reliability and validity of the Arabic version of the measures in a broader population are needed to enhance their applicability. As the study showed that female reported higher burnout level compared to male, studying psychosocial hazards Despite these limitations, the consistency of this study’s findings with existing literature strengthens its validity.

This study contributes to the existing literature by exploring the relationship between staff mental health and TAM. The findings revealed that factors related to TAM, specifically perceived usefulness and perceived ease of use, are significantly negatively correlated with burnout and depression. This study deepens understanding of technology acceptance, particularly in the UAE healthcare sector, by focusing on robotic pharmacies.

In addition, this study highlights the need to recognize the effects of technology on staff well-being. EHS leaders are encouraged to develop strategies to reduce stressors associated with advanced technologies (e.g., robotic pharmacies). These strategies should include maintaining a balanced workload and offering professional development opportunities for healthcare staff.

In conclusion, the current study explored differences in burnout, depression levels, and TAM among employees working in public hospitals in the UAE. Overall, automation had both positive and negative effects on workplace stressors experienced by pharmacy staff. It improved certain aspects, such as stress levels, workload allocation, and work-life balance, but also posed challenges, such as devaluing technician skills and increasing pressure on remaining staff. The findings indicate that sex plays a role in determining the level of technology acceptance and work-related well-being among pharmacists.

## Data Availability

The raw data supporting the conclusions of this article will be made available by the authors, without undue reservation.
